# Clinical patterns of vitiligo in Japan: A descriptive study using the JMDC claims database

**DOI:** 10.1111/1346-8138.17627

**Published:** 2025-01-20

**Authors:** Atsushi Tanemura, Yue Ma, Masayo Sakaki‐Yumoto, Shintaro Hiro, Tomohiro Hirose, Tamio Suzuki

**Affiliations:** ^1^ Department of Dermatology, Graduate School of Medicine Osaka University Osaka Japan; ^2^ Specialty Care Medical Affairs Japan Pfizer Japan Inc. Tokyo Japan; ^3^ Statistics Research & Data Science Pfizer R&D Japan Tokyo Japan; ^4^ Department of Dermatology, Faculty of Medicine Yamagata University Yamagata Japan

**Keywords:** autoimmune disease, comorbidity, Japan, pigmentation disorders, vitiligo

## Abstract

Vitiligo is a chronic autoimmune disorder that profoundly impacts patients' quality of life. Real‐world data on vitiligo in Japan are limited. This descriptive, cross‐sectional study used a claims database to evaluate vitiligo prevalence, patient demographics, treatments, and comorbidities in Japanese patients with vitiligo. Patients with claims for a vitiligo diagnosis in the JMDC database from January 2010 to December 2022 were included. Annual vitiligo prevalence, comorbidities, treatments, and medical facility visits were analyzed. Of 16 947 087 patients in the database during the 13‐year analysis period, a total of 26 358 patients (0.16%, 95% confidence interval 0.15–0.16) had a diagnosis of vitiligo. The standardized prevalence of vitiligo by sex and age in Japan remained generally consistent between 2010 (0.051%) and 2022 (0.056%). Atopic dermatitis was the most prevalent comorbidity. Comorbid atopic dermatitis prevalence increased between 2010 (21.8%) and 2022 (34.0%), and was highest among children aged 5–9 years. Other common comorbidities in 2022 included hypertension (10.4%), dyslipidemia (8.0%), anxiety disorder (7.4%), and psoriasis (7.0%). Topical corticosteroids were the most commonly used treatment throughout the period analyzed. Between 2010 and 2022, topical corticosteroid use decreased from 75.1% to 66.9%, and the use of narrowband ultraviolet B procedures increased from 19.2% to 28.1%. Mean duration of care was 12.9 months (standard deviation 20.5 months) and the median total number of outpatient medical facility visits was 3.0 (interquartile range 1.0–12.0). Key limitations include age and occupational biases in the JMDC database and potential misclassification of comorbidities due to off‐label treatment coding. Despite limitations in using a claims database, this study demonstrates consistent vitiligo prevalence in Japan, a high comorbidity burden, and evolving treatment patterns. Findings may guide clinical practice and treatment guidelines to improve management of vitiligo in Japanese patients.

## 1 INTRODUCTION

Vitiligo is a chronic autoimmune disease characterized by loss of pigment‐producing melanocytes that results in patchy white or depigmented areas on the skin.^1^ A recent systematic review of epidemiologic studies indicated a global lifetime prevalence of approximately 0.36% (95% credible interval 0.24–0.54), with estimates ranging from 0.19% to 0.52% in Western populations.^2^, ^3^ In 2016, the estimated prevalence of vitiligo in Asia was 0.1 (95% CI 0.1–0.2).^4^ Additionally, prevalence varies geographically from 0.23% (95% confidence interval [CI] 0.14–0.38) in East Asia to 0.52% (95% CI 0.33–0.82) in South Asia.^2^ In Japan, there is a scarcity of published data regarding vitiligo prevalence, but some estimates indicate this may range between 0.5% and 1.7%.^5^, ^6^

Vitiligo profoundly impacts patients' psychological well‐being and quality of life, particularly in women and individuals with darker skin tones, where the contrast between lesional and nonlesional skin is more pronounced.^1^, ^7^, ^8^, ^9^, ^10^ The visibility of depigmented patches often leads to feelings of stigmatization, self‐consciousness, embarrassment, and low self‐esteem.^10^, ^11^ Consequently, several psychiatric comorbidities, including depression, anxiety, emotional impairment, and suicidality, are common and may affect up to 50% of patients.^8^, ^11^, ^12^, ^13^ Vitiligo is also frequently associated with other comorbidities, including other autoimmune diseases and connective tissue and metabolic conditions that further complicate the management and general well‐being of affected individuals.^14^, ^15^, ^16^, ^17^, ^18^, ^19^ These comorbid conditions often cause further impairments in daily functioning and overall quality of life.^8^, ^10^, ^11^

The management recommendations for vitiligo endorsed by the International Vitiligo Task Force in 2023 underscore the importance of shared decision‐making and delineate specific treatment goals and modalities that aim to halt disease progression, stabilize depigmented lesions, and promote repigmentation.^20^ Topical treatment, phototherapy, or systemic treatment is suggested for active vitiligo, while maintenance treatment is recommended for stable vitiligo to prevent flares. Surgical techniques are reserved for vitiligo stable for at least 12 months without treatment.^20^, ^21^ The guidelines from the Japanese Dermatological Association (JDA), last published in 2013, recommend topical corticosteroids (TCS) as first‐line therapy for mild or moderate vitiligo present on 10%–20% of the body surface area.^22^

Due to outdated JDA guidelines and a lack of comprehensive, up‐to‐date, epidemiologic data on vitiligo in Japan, including sparse prevalence estimates and insufficient quantitative analyses of comorbidities and treatment patterns, there is a critical need for current evidence‐based insights to refine therapeutic strategies. To address these gaps and understand clinical needs, we conducted a real‐world study using data from a claims database to evaluate the prevalence of vitiligo, patient demographics, treatment patterns, and comorbidities in Japanese patients over a 13‐year period.

## 2 METHODS

### 2.1 Data source and study design

This descriptive, cross‐sectional study used the JMDC (formerly named as Japan Medical Data Center Co., Ltd.) claims database. The JMDC database is one of the largest claims databases in Japan and includes detailed inpatient, outpatient, and pharmacy claims data from Japan‐based health insurance associations for employees of large‐sized companies and their families. This longitudinal database does not include data from self‐employed individuals covered by the National Health Insurance program, employees of small‐ to middle‐sized companies, or government employees covered by mutual aid associations. Additionally, the JMDC primarily contains data from a young adult and middle‐aged population, as it predominantly covers employees and their families. Each patient can be tracked by a consistent unique identifier unless their health insurance changes. Based on a feasibility assessment in September 2023, data in the JMDC represented about 13.5% of the country's total population.

### 2.2 Participants and setting

Data were extracted from the JMDC claims database for the analysis period between January 1, 2010, and December 31, 2022. Patients were eligible to be included if they had at least one claim for a diagnosis of vitiligo (International Classification of Diseases, 10th Revision [ICD‐10] code: L80 [exclude senile], D229 [Sutton's halo nevus]) during the analysis period. The index month was defined as the month of the first record of the vitiligo diagnosis within the analysis period for each assessment (Supporting Information Figure S1). Patients diagnosed before 2010 who did not visit a clinic or hospital between 2010 and 2022 were not included in the study. For each vitiligo patient, their unique identifier was used to track their comorbidities, treatments, and medical facility visit activities up to the date of their last recorded visit.

Data were included for patients with at least one claim for a diagnosis of vitiligo in a medical facility located within a government‐designated city (either ordinance‐designated cities [per the Ministry of Internal Affairs and Communications; Supporting Information Table S1] or other cities). The clinical department where patients received their initial vitiligo diagnosis was categorized based on the bed capacity of the medical institution: those with fewer than 20 beds were classified as clinics, while those with 20 or more beds were designated as hospitals. Subsequently, four categories were established: dermatology clinic, dermatology hospital, other clinic, and other hospital, reflecting the size and specialization of the medical institutions.

### 2.3 Outcomes

The extracted data included age at index month, sex, medical diagnoses, clinical procedures, medications prescribed, and medical facility visit activities.

#### 2.3.1 Prevalence of vitiligo

Overall prevalence was defined as the proportion of patients with at least one claim for a diagnosis of vitiligo (L80 [exclude senile]) during the analysis period among those included in the JMDC for at least 1 month from 2010 to 2022. Annual vitiligo prevalence in Japan was calculated as the proportion of patients with a diagnosis code for vitiligo at least once during a given year among those who were included in the JMDC for at least 1 month that year. Standardized annual prevalence proportions were calculated based on national population data by age–sex matching to account for the fluctuation in age and sex proportions in the JMDC population relative to the national population. The national population data were provided by the Ministry of Internal Affairs and Communications HP. Monthly prevalence of vitiligo was calculated for the years 2010, 2015, 2019, and 2022. Monthly prevalence was defined as the proportion of patients with at least one claim for a diagnosis of vitiligo in each month in each target year among those enrolled in the JMDC database in each month in each target year.

#### 2.3.2 Prevalence of comorbidities

A 12‐month period (6 months before the index month and the index month plus 6 months) was defined as the target period to assess the comorbidities in patients with vitiligo. Autoimmune/inflammatory, metabolic and end‐organ, and psychiatric comorbidities were evaluated (Supporting Information Table S2). Patients with at least one claim for one of the definitive ICD‐10 diagnosis codes during the period of 6 months prior to and after the index month were included. The proportion of patients with comorbidities was evaluated annually. A subgroup analysis was performed to evaluate the proportion of patients with comorbidities by age, categorized in 5‐year intervals for those aged 0–59 years, and a single category for those aged 60–74 years.

#### 2.3.3 Treatment patterns

A 12‐month period beginning on or after the index month for vitiligo was defined as the target period to assess treatment patterns for patients who had at least one claim for a diagnosis of vitiligo for the target years of 2010, 2015, 2019, and 2022. If a patient received multiple types of treatment during the target period, each treatment type was counted independently, resulting in potential multiple counts for a single patient. Treatments and medical procedures evaluated are summarized in Supporting Information Tables S3 and S4, respectively. A subgroup analysis was carried out to evaluate treatment patterns by age group in 2022.

#### 2.3.4 Medical facility visit activities

The duration of vitiligo treatment (months) was calculated as the time from the first month of vitiligo diagnosis to the last recorded medical facility visit for vitiligo, plus 1 month. Cumulative outpatient medical facility visits were calculated by counting the number of outpatient visits with vitiligo during the vitiligo treatment period across medical facilities. The number of medical facilities visited (single or multiple) during the treatment period was also analyzed. In the JMDC database, only the primary department of a hospital is recorded, even when multiple departments may be present.

### 2.4 Statistical analysis

Descriptive statistics including mean, standard deviation (SD), median, range (minimum to maximum), and interquartile range (IQR) were used to summarize the data. Confidence intervals (CIs) were calculated at a confidence level of 95% using the Clopper–Pearson method. Prevalence proportion (percentage) was measured as (number of cases/total population) × 100. Missing values were not imputed. All statistical analyses were performed using Amazon Redshift (version 1.0) and SAS (version 9.4 TS1M6).

## 3 RESULTS

### 3.1 Patient characteristics

Of 16 947 087 individuals with data in the JDMC database between June 2010 and December 2022, 26 358 patients (0.16%, 95% CI 0.15–0.16) met the criteria for vitiligo diagnosis and were included in the study population (Table 1). A total of 26 358 patients had a diagnosis of vitiligo only (ICD‐10 code L80 [exclude senile]), 214 patients had a diagnosis of Sutton's halo nevus only (ICD‐10 code D229), and 107 patients had both vitiligo and Sutton's halo nevus diagnoses. The mean age at the time of vitiligo diagnosis was 30.8 years (SD 20.9 years) and 53.6% of patients were female. Most patients (67.0%) were diagnosed in a dermatology clinic.

**TABLE 1:** Characteristics of patients with a diagnosis of vitiligo at index month.

Characteristic	*N* = 26 358
Sex, *n* (%)	
Male	12 240 (46.4)
Female	14 118 (53.6)
Age, years	
Mean (SD)	30.8 (20.9)
Median (IQR)	32.0 (10.0–49.0)
Range, min–max	0–74
Age group, *n* (%)	
0–9 years	6146 (23.3)
10–19 years	3813 (14.5)
20–29 years	2538 (9.6)
30–39 years	3531 (13.4)
40–49 years	4077 (15.5)
50–59 years	3783 (14.4)
60–74 years	2470 (9.4)
Medical facility at first diagnosis of vitiligo, *n* (%)^a^	
Dermatology clinic	17 660 (67.0)
Dermatology hospital	0 (0.0)
Other clinic	4775 (18.1)
Other hospital	3923 (14.9)
Division of local government, *n* (%)	
Ordinance‐designated city	6841 (26.0)
Other cities	19 517 (74.0)

Abbreviations: IQR, interquartile range; SD, standard deviation.

^a^ Facilities with fewer than 20 beds were classified as clinics, while those with 20 or more beds were designated as hospitals. Subsequently, four categories were established: dermatology clinic, dermatology hospital, other clinic, and other hospital, reflecting the size and specialization of the medical institutions. A dermatology hospital is defined as a hospital with more than 20 beds where the primary department is dermatology.

### 3.2 Prevalence of vitiligo

**FIGURE 1:**
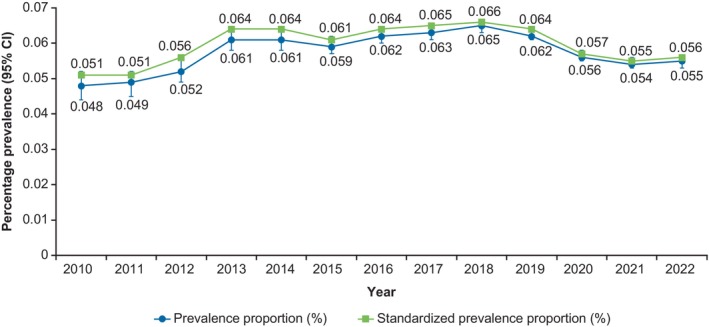
Annual prevalence of vitiligo for patients aged ≤65 years between 2010 and 2022, according the JMDC claims database. CI, confidence interval.

The number of individuals with data in the JMDC increased from 1 028 999 in 2010 to 10 820 810 in 2022. The standardized annual prevalence of vitiligo was 0.051% in 2010 and increased to 0.064% in 2013; thereafter, annual prevalence remained consistent until 2020, when there was a slight decrease to 0.056% (Figure 1). The prevalence of vitiligo among patients aged 0–12 years and 13–19 years increased between 2010 and 2022, while annual prevalence among patients aged ≥20 years remained generally consistent, with a slight increase between 2012 and 2014. Compared with children in other age groups, children aged 0–12 years had the highest prevalence of vitiligo overall and the greatest increase in annual prevalence between 2010 and 2022 (Supporting Information Figure S2). In terms of seasonality, the prevalence of vitiligo was generally higher during the period between July through October and was relatively consistent throughout the rest of the year (Supporting Information Figure S3).

### 3.3 Prevalence of comorbidities

**FIGURE 2:**
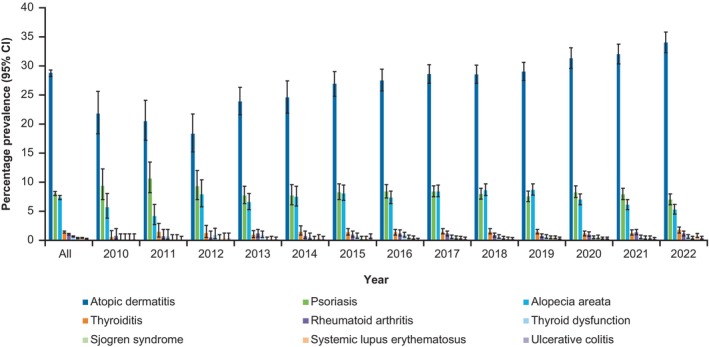
Annual prevalence of autoimmune/inflammatory comorbidities in patients with vitiligo in the JMDC database between 2010 and 2022 (*N* = 26 358). CI, confidence interval.

**FIGURE 3:**
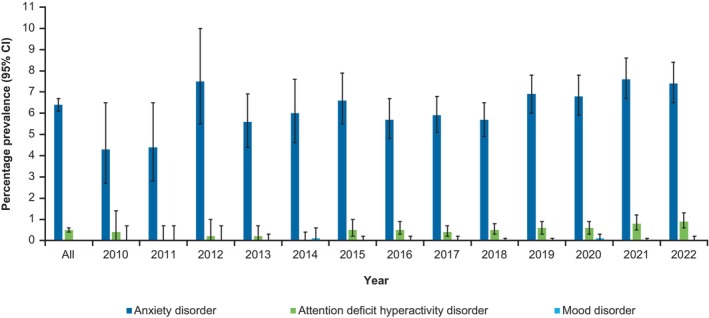
Annual prevalence of psychiatric comorbidities in patients with vitiligo in the JMDC database between 2010 and 2022 (*N* = 26 358). CI, confidence interval.

**FIGURE 4:**
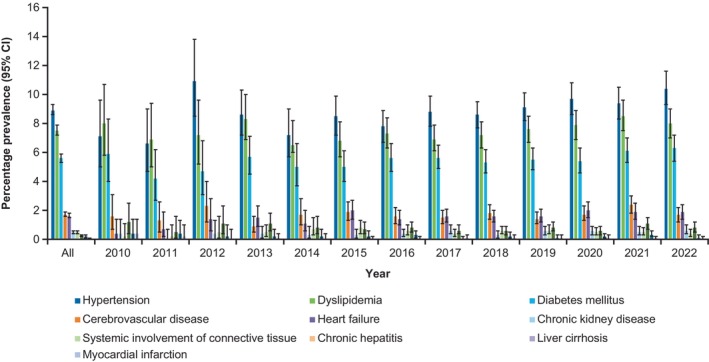
Annual prevalence of metabolic and end‐organ comorbidities in patients with vitiligo in the JMDC database between 2010 and 2022 (*N* = 26 358). CI, confidence interval.

Among all patients with vitiligo, atopic dermatitis (AD), psoriasis, and alopecia areata (AA) were the most prevalent autoimmune/inflammatory comorbidities throughout the analysis period, with annual prevalence estimates of 34.0%, 7.0%, and 5.3%, respectively, in 2022 (Figure 2). The annual prevalence of comorbid AD increased from 21.8% in 2010 to 34.0% in 2022. The prevalence of comorbid lupus erythematosus, Crohn's disease, Behçet's disease, albinism, and dermatomyositis were each ≤0.2% from 2010 to 2022. In a subgroup analysis of comorbidities by age, the prevalence of comorbid AD was highest among patients aged 5–9 years (39.4%) and lowest among patients aged 60–74 years (20.2%; Supporting Information Figure S4). The prevalence of psoriasis was 4.0% in patients aged 0–4 years and generally increased with age; 11.0% of patients aged 60–74 years had psoriasis. Anxiety disorder was the most common psychiatric comorbidity in patients with vitiligo throughout the analysis period and increased in prevalence from 4.3% in 2010 to 7.4% in 2022 (Figure 3). Among patients with vitiligo and anxiety, 12.3% had comorbid AA (data not shown). The most prevalent metabolic and end‐organ comorbidities throughout the analysis period were hypertension, dyslipidemia, and diabetes mellitus, with estimates of 10.4%, 8.0% and 6.3%, respectively, in 2022 (Figure 4). The prevalence of myasthenia gravis, Addison's disease, and pernicious anemia was ≤0.2% fr each between 2010 and 2022.

### 3.4 Treatment patterns

**FIGURE 5:**
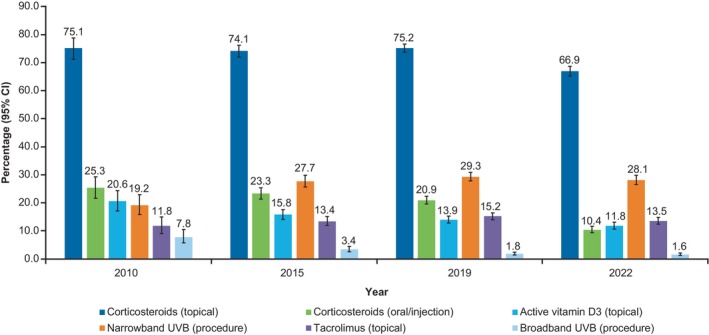
Treatments used for vitiligo in 2010, 2015, 2019, and 2022 (*N* = 26 358). CI, confidence interval; UVB, ultraviolet B.

TCSs were the most common vitiligo treatment used in this population, but their usage decreased from 75.1% in 2010 to 66.9% in 2022 (Figure 5). In the total population with vitiligo, oral/injectable corticosteroid use also declined from 25.3% in 2010 to 10.4% in 2022, and the use of narrowband ultraviolet B (NB‐UVB) increased from 19.2% in 2010 to 28.1% in 2022. The use of psoralen and ultraviolet A (PUVA), skin grafting, and immunosuppressants (oral/injection) was <5% throughout the analysis period. TCSs were the most common vitiligo treatment across all age groups (66.9% in 2022), followed by NB‐UVB (28.1% in 2022) (Supporting Information Figure S5). Treatment use was generally similar across age groups in 2022.

### 3.5 Medical facility visit activities

The mean and median duration of care were 12.9 months (SD, 20.5) and 3.0 months (IQR 1.0–16.0), respectively (Table 2). The median total number of outpatient medical facility visits was 3.0 (IQR 1.0–12.0). Most patients (84.6%) visited a single medical facility.

**TABLE 2:** Medical facility visit activities among patients with vitiligo.

	*N* = 26 358
Duration of care, months	
Mean (SD)	12.9 (20.5)
Median (IQR)	3.0 (1.0–16.0)
Min–max	1–156
Outpatient medical facility visits	
Mean (SD)	14.7 (35.1)
Median (IQR)	3.0 (1.0–12.0)
Min–max	1–827
Medical facility utilization, *n* (%)	
Single medical facility	22 288 (84.6)
Multiple medical facilities	4070 (15.4)

Abbreviations: IQR, interquartile range; SD, standard deviation.

## 4 DISCUSSION

The objective of this study was to assess the prevalence, comorbidities, and treatment patterns of vitiligo in Japanese patients using data from the JMDC claims database over the 13‐year period from 2010 to 2022. Our analysis provides the first detailed examination of vitiligo epidemiology in Japan, revealing consistent prevalence rates, a significant comorbidity burden, and evolving treatment use among affected individuals. Findings from the current study involving a large sample size offer robust and comprehensive insights for healthcare planning efforts that aim to address the care needs of Japanese patients with vitiligo.

The overall prevalence of vitiligo during the analysis period was 0.15%, which is lower than estimates from other studies covering the same time period. A nationwide, cross‐sectional, multicenter, hospital‐based study in Japan published in 2009 reported a vitiligo prevalence of 1.68% (*n*/*N*, 1134/67 448).^5^ The higher prevalence observed in that study may be due to selection bias because it included only patients who visited dermatology clinics or hospitals. A small population‐based survey conducted online in 2022 reported a prevalence of 0.54% (*n*/*N*, 45/8392) for Japanese individuals who provided self‐reported data.^6^ This survey may have included individuals with vitiligo signs who were not formally diagnosed by a physician. In contrast, the current analysis included patients diagnosed across all levels of care and only physician‐diagnosed patients, which could account for the lower observed prevalence.

In this study population, females represented a slightly higher proportion of patients with vitiligo compared with males, which is consistent with previous studies in Asian patients or patients with darker Fitzpatrick skin types.^4^, ^18^, ^23^ Several studies have shown that females tend to experience lower quality of life and more pronounced psychopathological symptoms of vitiligo compared with males.^24^ Additionally, women with vitiligo frequently perceive themselves as lacking physical attractiveness.^24^ Findings from previous studies suggest that women face greater challenges with vitiligo and may be more inclined to seek treatment due to the stricter and more pervasive appearance norms imposed on them compared to men.^25^

Annual standardized prevalence of vitiligo was relatively stable throughout the analysis period, with minor fluctuations. The increase in standardized prevalence from 0.051% in 2010 to 0.064% in 2013 may be attributed to rhododendrol‐induced vitiligo from a skin‐whitening cosmetic launched in Japan in 2008.^26^ In a survey conducted by the marketing authorization holder, 19 606 consumers reported developing leukoderma after using 2% rhododendrol‐containing cosmetics,^27^ therefore misdiagnosis of chemical vitiligo as vitiligo could have contributed to the slight increase in prevalence observed in the current study from 2013. Informational campaigns, including newspaper articles and broadcast programs, about rhododendrol‐induced vitiligo may have also raised awareness of vitiligo as a medical condition and led to a higher consulting rate of patients with physicians. The decrease in prevalence in 2020 may reflect the impact of COVID‐19, which reduced the capacity of non‐urgent and specialized medical services and the number of people seeking non‐urgent medical care.^28^

The annual prevalence of vitiligo was highest in children aged 0–12 years compared with other age groups, whereas a recent systematic review, which assessed lifetime prevalence, found higher rates in adults than in children across all geographic regions.^2^ This difference likely arises from the distinct study designs; our data capture the current distribution of vitiligo within a year, while the review reflects the overall lifetime burden, which tends to be higher in adults due to the longer duration of time over which vitiligo can develop. Age of onset is bimodal, consisting of early‐ and late‐onset vitiligo subgroups.^29^ Early onset in children may be associated with a family history of the disease.^29^, ^30^

Our study found that comorbidities were frequent among patients with vitiligo, which aligns with previous research.^11^, ^14^, ^15^ The prevalence of AD increased from 21.8% in 2010 to 34.0% in 2022. This upward trend suggests a growing overlap between vitiligo and atopic conditions, which is consistent with other studies and indicates a heightened T‐helper 2 immune response in patients with vitiligo.^14^, ^31^ Additionally, vitiligo shares inflammatory pathways with conditions like alopecia and psoriasis, which were also prevalent in the current study, with overlapping immune mechanisms implicated in their co‐occurrence.^15^ IL‐17 has emerged as a key signaling molecule in the pathogenesis of vitiligo, AD, psoriasis, and AA.^15^ The observed high prevalence of AD and psoriasis in the JMDC claims database may not be reflective of a direct epidemiological association. In clinical practice in Japan, diagnosis codes of AD and psoriasis may in some cases be used to facilitate the use of off‐label treatments for vitiligo, potentially inflating the reported prevalence. Additionally, the high prevalence of comorbid AD and psoriasis may be influenced by an overlap in treatments, with topical corticosteroids and tacrolimus used for both AD and vitiligo, and topical active vitamin D3 used for both psoriasis and vitiligo, potentially leading to over‐reporting of these conditions as comorbidities.

Findings also highlight a significant burden of metabolic syndrome in the Japanese population, a trend observed in various cohorts worldwide.^15^, ^32^, ^33^ A growing body of evidence suggests an association between the systemic inflammation characteristic of vitiligo and metabolic dysregulation.^15^ Notably, proinflammatory cytokines such as interleukin (IL)‐6, IL‐1, and tumor necrosis factor‐α, known contributors to insulin resistance and endothelial dysfunction in metabolic syndrome, are also implicated in vitiligo pathogenesis.

Comorbid anxiety was the most prevalent psychiatric comorbidity in this population, which mirrors global trends.^8^, ^11^, ^12^ The prevalence of anxiety may be underestimated, as the JMDC claims database includes only diagnosed anxiety cases and individuals with psychiatric comorbidities in Japan may not seek outpatient mental health care. Among patients with vitiligo and anxiety, only 12.3% had comorbid AA, suggesting that the prevalence of anxiety may be numerically greater in patients with vitiligo without AA than in those with both vitiligo and AA. These findings are surprising because multimorbidity is typically linked with poorer mental health and reduced quality of life.^34^ AA is known to have a significant negative impact on the emotional and psychological wellbeing of those affected.^35^ The co‐occurrence of vitiligo and AA may profoundly affect a patient's appearance due to depigmentation and hair loss, potentially exacerbating quality of life impairments and the psychological burden of managing multiple visible or stigmatizing conditions. It is noteworthy that during the analysis period no established treatment specifically addressing the concurrent management of vitiligo and AA was available. The prevalence of anxiety among patients with vitiligo with comorbid AD or psoriasis was not assessed because these conditions could have been proxy diagnoses for off‐label vitiligo treatments, as noted above. Findings highlight the need for integrated care approaches that address both the dermatological disease and comorbidity burden among patients with vitiligo.

This study provides comprehensive insights into the treatment landscape for vitiligo in Japan. Corticosteroids, including both topical and oral/injectable formulations, and phototherapy were commonly employed treatments throughout the analysis period and across different age groups. These findings mirror the JDA guidelines during that time, which recommended TCS as first‐line therapy for vitiligo.^22^ More recent guidelines from the International Vitiligo Task Force published in 2023 recommend TCS for vitiligo treatment, particularly for extrafacial and limited areas, but advise caution for prolonged use, especially in children, to avoid systemic absorption and adverse effects.^21^ The observed decline in the frequency of corticosteroid use between 2010 and 2022 may reflect evolving treatment practices possibly driven by safety concerns associated with corticosteroid use.^21^

As expected, most patients were diagnosed in a dermatology clinic because patients are rarely referred to hospital dermatology departments in Japan. The mean duration of care for vitiligo was 12.9 months and the median was only 3.0 months, indicating that most patients had brief care periods. Despite the chronic nature of vitiligo and high relapse rate requiring long‐term management,^36^ many patients visited medical facilities only a few times, with a median of three visits. The discrepancy between mean and median durations of care may result from a few patients receiving long‐term care, such as those undergoing NB‐UVB treatment. NB‐UVB was the second most frequently used treatment in the current study and, according to global treatment guidelines, requires regular treatment sessions, often two or three times per week for several months.^21^ However, the use of NB‐UVB may have been infrequent or many patients may have discontinued treatment shortly after diagnosis due to the lack of a definitive cure for vitiligo. The higher mean duration of care may also reflect the inclusion of patients who revisited medical facilities years after their initial diagnosis, thereby inflating the mean duration of care.

A strength of this study is that the JMDC claims database afforded a substantial sample size of patients with vitiligo (*n* = 26 358) drawn from the general population who accessed medical services. It is important to note that the JMDC database has grown significantly, from representing only 1% of the Japanese population initially to nearly 10% in 2022, which may enhance the accuracy of vitiligo prevalence estimates in recent years. The increase in JMDC database coverage over 13 years likely reflects structural changes, such as the addition of new insurance associations and expanded data sources, rather than solely an increase in claims data. A notable advantage of the JMDC database is its provision of a unique and consistent identification number for each patient that facilitated comprehensive patient tracking even after a patient visited a different medical facility. Additionally, to address potential cases of undiagnosed vitiligo, the study included patients with Sutton's halo nevus, a condition that can co‐occur with vitiligo.^37^ The prevalence of this condition was low in the JMDC population, further reinforcing the robustness of our data. However, the JMDC database is limited by underrepresentation of individuals aged ≥60 years and by the fact that its coverage mainly includes those with employer‐provided health insurance in mid‐ to large‐sized companies and their families, thereby excluding individuals in smaller firms and specific occupational groups.

In conclusion, this large‐scale study utilizing real‐world data from the JMDC claims database provides significant insights into vitiligo trends in Japan over the 13‐year period. Despite inherent limitations in the database, our findings underscore a consistent prevalence of vitiligo and highlight the substantial burden of comorbidities associated with the condition. Moreover, the relatively short duration of care observed in this population suggests a potential need for enhanced treatment strategies that offer ongoing care and follow‐up. Overall, these findings can inform clinical practice and guide the development of personalized treatment approaches aimed at improving the management of vitiligo in Japan.

## FUNDING INFORMATION

This study, including the extraction, analysis, and interpretation of data and writing of the report, was sponsored by Pfizer Japan Inc., Tokyo, Japan.

## CONFLICT OF INTEREST STATEMENT

A.T. reported no conflict of interest. T.S. is an Editorial Board member of *Journal of Dermatology* and a co‐author of this article. To minimize bias, they were excluded from all editorial decision‐making related to the acceptance of this article for publication. T.S. has served as a consultant for AbbVie, Pfizer, J‐TEC and Pola; conducted joint research with Kao Corporation and Maruho Co., Ltd.; and is the secretary general of the Japanese Society of Vitiligo. M.S.‐Y., T.H., and Y.M. are employees of Pfizer Japan Inc. and stockholders of Pfizer Inc. S.H. is an employee of Pfizer R&D Japan GK and a stockholder of Pfizer Inc.

## ETHICS STATEMENT

Approval of the research protocol by an Institutional Reviewer Board: Institutional review board approval and ethics committee approval were not required. This study was conducted in accordance with legal and regulatory requirements, as well as with scientific purpose, value, and rigor, and followed generally accepted research practices as described in the Guidelines for Good Pharmacoepidemiology Practices issued by the International Society for Pharmacoepidemiology.

Informed Consent: This study used an anonymized structured database that did not contain any personal information that allowed for identification of individuals. According to the Ethical Guidelines for Human Life Science and Medical Research in Japan, informed consent is not required for studies using anonymized data.

Registry and the Registration No.: N/A.

Animal Studies: N/A.

## Supporting information


Data S1.

## Data Availability

Upon request, and subject to review, Pfizer will provide the data that support the findings of this study. Subject to certain criteria, conditions, and exceptions, Pfizer may also provide access to the related individual de‐identified participant data. See https://www.pfizer.com/science/clinical‐trials/trial‐data‐and‐results for more information.
