# Supplementary material for: Clinical patterns of vitiligo in Japan: A descriptive study using the JMDC claims database
Source: J Dermatol. 2025 Jan 20;52(5):831–40. doi: 10.1111/1346-8138.17627 (PMC12056275; doi:10.1111/1346-8138.17627)

# **Supporting Information**

**Clinical Patterns of Vitiligo in Japan: A Descriptive Study Using the JMDC Claims Database**

Atsushi Tanemura,^1^* Yue Ma,^2^* Masayo Sakaki-Yumoto,^2^ Shintaro Hiro,^3^ Tomohiro Hirose,^2^

Tamio Suzuki^4^

^1^Department of Dermatology, Graduate School of Medicine, Osaka University, Osaka, Japan; ^2^ Medical Affairs Japan, Pfizer Japan Inc., Tokyo, Japan; ^3^Statistics Research & Data Science, Pfizer R&D Japan, Tokyo, Japan; ^4^Department of Dermatology, Faculty of Medicine, Yamagata University, Yamagata, Japan

*These authors contributed equally and are co-lead authors of this manuscript.

**Corresponding author**

Tomohiro Hirose

Specialty Care Medical Affairs

Pfizer Japan Inc.

Shinjuku Bunka Quint Bldg.

3-22-7, Yoyogi,

Shibuya-ku, Tokyo

151-8589 Japan

Email: [tomohiro.hirose@pfizer.com](mailto:tomohiro.hirose@pfizer.com)

Telephone: +81 90 1250 7282

**Supporting Information Table S1. Ordinance-designated cities and population.**

| **City** | **Population*** |
| --- | --- |
| Osaka | 2,752,412 |
| Nagoya | 2,332,176 |
| Kyoto | 1,463,723 |
| Yokohama | 3,777,491 |
| Kobe | 1,525,152 |
| Kitakyushu | 939,029 |
| Sapporo | 1,973,395 |
| Kawasaki | 1,538,262 |
| Fukuoka | 1,612,392 |
| Hiroshima | 1,200,754 |
| Sendai | 1,096,704 |
| Chiba | 974,951 |
| Saitama | 1,324,025 |
| Shizuoka | 693,389 |
| Sakai | 826,161 |
| Niigata | 789,275 |
| Hamamatsu | 790,718 |
| Okayama | 724,691 |
| Sagamihara | 725,493 |
| Kumamoto | 738,865 |

*Population figures are from the 2020 census (confirmed figures).

**Supporting Information Table S2. Patient comorbidities and ICD-10 codes.**

| **Variable** | **ICD-10 code** |
| --- | --- |
| **Autoimmune/Inflammatory diseases^a^** |  |
| Alopecia areata | L63 |
| Atopic dermatitis | L20 |
| Psoriasis | L40, L41 |
| Crohn’s disease | K50 |
| Ulcerative colitis | K51 |
| Rheumatoid arthritis | M05, M06, M080 |
| Systemic lupus erythematosus | M32 |
| Behcet disease | M352 |
| Sjögren syndrome | M350 |
| Albinism | E703 |
| Lupus erythematosus | L93 |
| Dermatomyositis | M330, M331, M339 |
| Thyroiditis | E060, E061, E063, E064, E065, E069 |
| Thyroid dysfunction | E079 |
| **Metabolic and end-organ diseases^a^** |  |
| Hypertension | I10-I15 |
| Diabetes mellitus | E10-E14 |
| Pernicious anemia | D510 |
| Addison's disease | E271 |
| Myasthenia gravis | G700 |
| Systemic involvement of connective tissue | M359 |
| Chronic hepatitis C | B182 |
| Dyslipidemia | E785 |
| Myocardial infarction | I21-I22 |
| Heart failure | I50 |
| Cerebrovascular disease | I60-I69 |
| Chronic hepatitis | K73 |
| Liver cirrhosis | K74 |
| Chronic kidney disease | N18 |
| **Psychiatric disease ^a^** |  |
| Anxiety disorder | F40, F41, F42, F43, F44, F45, F48 |
| Attention deficit hyperactivity disorder | F90, F988 |
| Mood disorder | F39, F349, F381 |

ICD, International Classification of Diseases, 10th Revision.

**^a^**At least one definitive diagnosis of the ICD-10 code during the period of 6 months prior to and after the index month.

**Supporting Information Table S3. Vitiligo treatments and ATC codes.**

| **ATC Code** | **ATC Name** | **Ingredient Name** | **Dosage Form** |
| --- | --- | --- | --- |
| D07A | Topical corticosteroids, plain | Amcinonide; alclometasone propionate; clobetasone butyrate; clobetasol propionate ester; diflucortolone valerate ester; difluprednate; diflorasone acetate; dexamethasone; dexamethasone propionate; dexamethasone valerate; deprodone propionate ester; triamcinolone acetonide; halcinonide; hydrocortisone acetate; hydrocortisone butyrate; fluocinonide; fluocinolone acetonide; fludroxycortide; flumetasone pivalate; prednisolone; prednisolone valerate acetate; beclometasone propionate; betamethasone dipropionate; betamethasone valerate; betamethasone butyrate propionate; mometasone furoate ester; hydrocortisone butyrate propionate | Topical |
| D07B1 | Topical corticosteroids with antibiotics | Oxytetracycline hydrochloride/hydrocortisone; chloramphenicol/fradiomycin sulfate/prednisolone; tetracycline hydrochloride/hydrocortisone acetate; triamcinolone acetonide, fradiomycin sulfate & gramicidin; hydrocortisone acetate/fradiomycin sulfate/diphenhydramine hydrochloride; fradiomycin sulfate/prednisolone; fluocinolone acetonide/fradiomycin sulfate; betamethasone valerate/gentamicin sulfate; betamethasone valerate/fradiomycin sulfate; killed Escherichia coli, Staphylococcus, Streptococcus, Pseudomonas & hydrocortisone | Topical |
| D07B4 | Other topical corticosteroid combinations | Glycyrrhetinic acid, hydrocortisone acetate & pyridoxine; dexamethasone, glycyrrhetinic acid/pyridoxine hydrocortisone crotamiton | Topical |
| D05A | Topical antipsoriatics | Calcipotriol; tacalcitol; maxacalcitol; calcipotriol hydrate/betamethasone dipropionate; maxacalcitol/betamethasone butyrate dipropionate | Topical |
| D05X | Other topical non-steroid preparations for inflammatory skin conditions | Tacrolimus hydrate | Topical |
| D11A | Other dermatological preparations | Methoxsalen | Topical |
|  |  | Methoxsalen | Oral |
| H02A1 | Injectable corticosteroid monotherapy | Dexamethasone palmitate; dexamethasone sodium metasulfobenzoate; dexamethasone sodium phosphate; dexamethasone acetate; triamcinolone; triamcinolone acetonide; halopredone acetate; hydrocortisone sodium succinate; hydrocortisone sodium phosphate; prednisolone sodium succinate; prednisolone sodium phosphate; betamethasone sodium phosphate; betamethasone acetate/betamethasone sodium phosphate; methylprednisolone sodium succinate; methylprednisolone acetate | Injection |
| H02A2 | Oral corticosteroid monotherapy | Cortisone acetate; dexamethasone; triamcinolone; paramethasone acetate; hydrocortisone; fludrocortisone acetate; prednisolone; betamethasone; methylprednisolone | Oral |
| H02B | Systemic corticosteroid combination | Betamethasone/D-chlorpheniramine maleate | Oral |
| L01B | Metabolic antagonist | Methotrexate | Injectable |
|  |  | Methotrexate | Oral |
| L04X | Other immunosuppressants | Cyclosporine; tacrolimus hydrate | Injectable |
|  |  | Azathioprine; apremilast; upadacitinib hydrate; cyclosporine; tacrolimus hydrate; baricitinib | Oral |
| M01C | Specific anti-rheumatic drug | Methotrexate | Injectable |
|  |  | Methotrexate | Oral |

ACT, Anatomical Therapeutic Chemical.

**Supporting Information Table S4. Vitiligo medical procedures and point codes and medical procedure codes.**

| **Point code** | **Medical procedure code** | **Medical procedure name** |
| --- | --- | --- |
| J054 | 140028810 | Dermatological phototherapy (infrared or UV) |
|  | 140051510 | Dermatological phototherapy (medium wave UV, 308-313 nm) |
|  | 140029010 | Dermatological phototherapy (long wave or medium wave UV) |
|  | 140029010 | Dermatological phototherapy (long wave/medium wave UV, 290-315 nm) |
| K013 | 150006410 | Full thickness or split thickness skin graft (<25 cm²) |
|  | 150006410 | Full thickness or split thickness skin graft (<25 cm²) |
|  | 150006410 | Split thickness skin graft (<25 cm²) |
|  | 150006510 | Full thickness or split thickness skin graft (25-100 cm²) |
|  | 150006510 | Full thickness or split thickness skin graft (25-100 cm²) |
|  | 150006510 | Split thickness skin graft (25-100 cm²) |
|  | 150006610 | Full thickness or split thickness skin graft (<200 cm²) |
|  | 150006610 | Full thickness or split thickness skin graft (100-200 cm²) |
|  | 150006610 | Split thickness skin graft (100-200 cm²) |
|  | 150006710 | Full thickness or split thickness skin graft (>200 cm²) |
|  | 150006710 | Full thickness or split thickness skin graft (>200 cm²) |
|  | 150006710 | Split thickness skin graft (>200 cm²) |
|  | 150007250 | Reverdin skin graft (<25 cm²) |
|  | 150007350 | Reverdin skin graft (25-100 cm²) |
|  | 150007450 | Reverdin skin graft (100-200 cm²) |
|  | 150007550 | Reverdin skin graft (>200 cm²) |
|  | 150442750 | Split thickness skin graft (<25 cm², autologous skin cell transplantation) |
|  | 150442850 | Split thickness skin graft (25-100 cm², autologous skin cell transplantation) |
|  | 150442950 | Split thickness skin graft (100-200 cm², autologous skin cell transplantation) |
|  | 150443050 | Split thickness skin graft (>200 cm², autologous skin cell transplantation) |
| K013-2 | 150333710 | Full thickness skin graft (<25 cm²) |
|  | 150333810 | Full thickness skin graft (25-100 cm²) |
|  | 150333910 | Full thickness skin graft (100-200 cm²) |
|  | 150334010 | Full thickness skin graft (>200 cm²) |
| K014 | 150007610 | Skin transplantation |
|  | 150007610 | Skin transplantation (living/cultured) |
|  | 150007770 | Living skin graft add-on |
|  | 150007770 | Living skin graft (donor's convalescence costs) |
|  | 150007770 | Living skin graft (donor's convalescence costs) add-on |

UV, ultraviolet.

**Supporting Information Figure S1. Study design.**


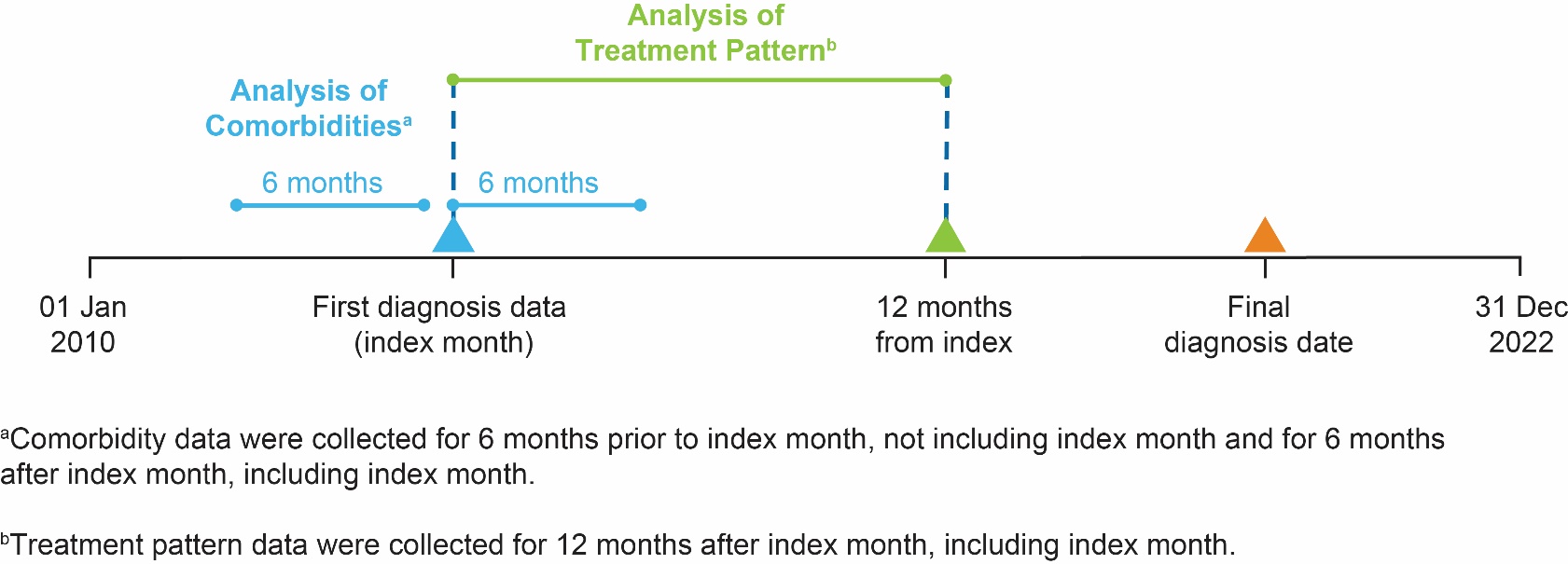


**Supporting Information Figure S2. Annual prevalence of vitiligo in the JMDC claims database between 2010 and 2022, stratified by age (N = 26,358).**


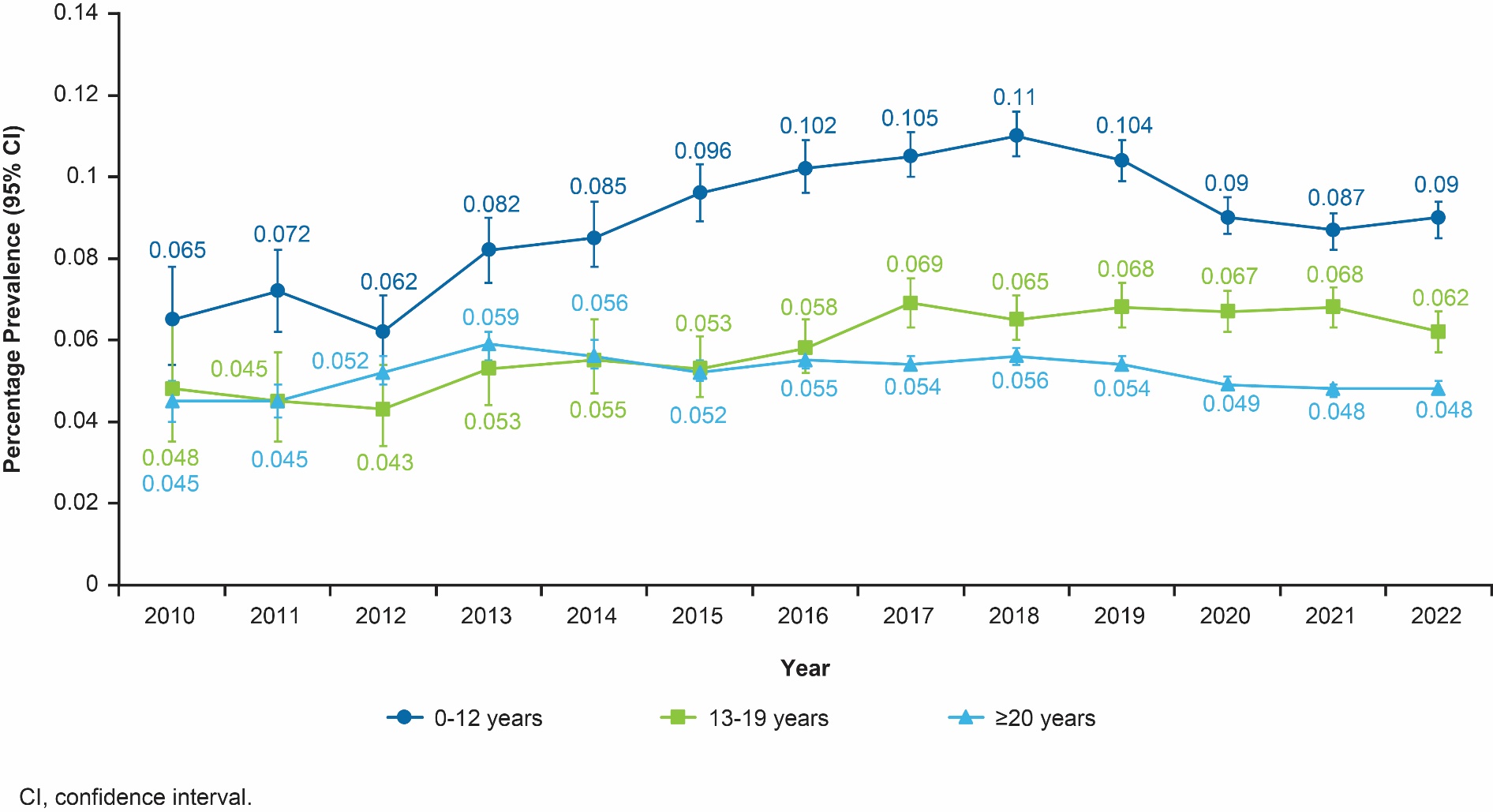


**Supporting Information Figure S3. Monthly prevalence of vitiligo in the JMDC claims database in 2010, 2015, 2019, and 2022 (N = 26,358).**


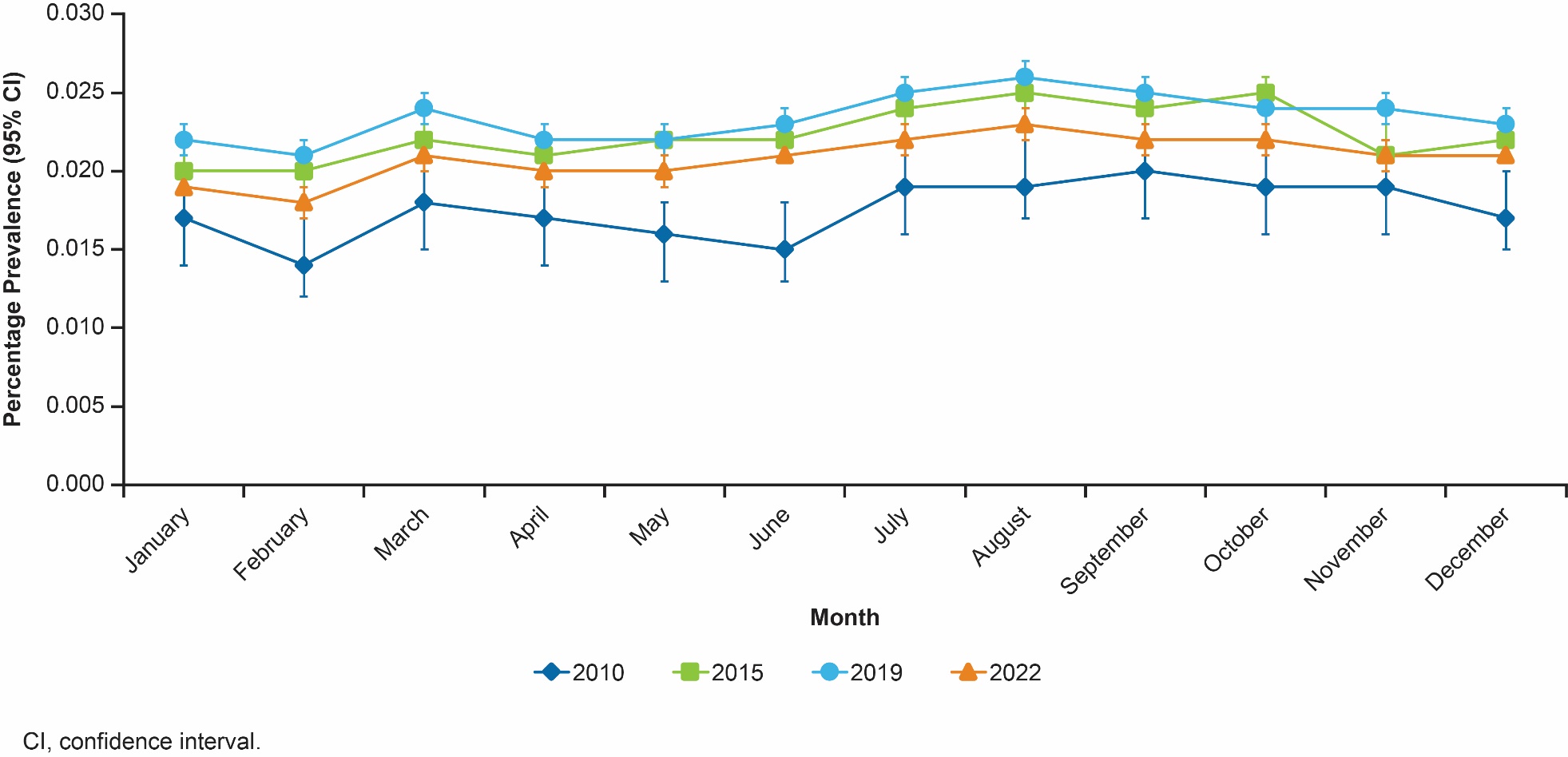


**Supporting Information Figure S4.Prevalence of comorbidities in patients with vitiligo in the JMDC database, stratified by age (N = 26,358).**


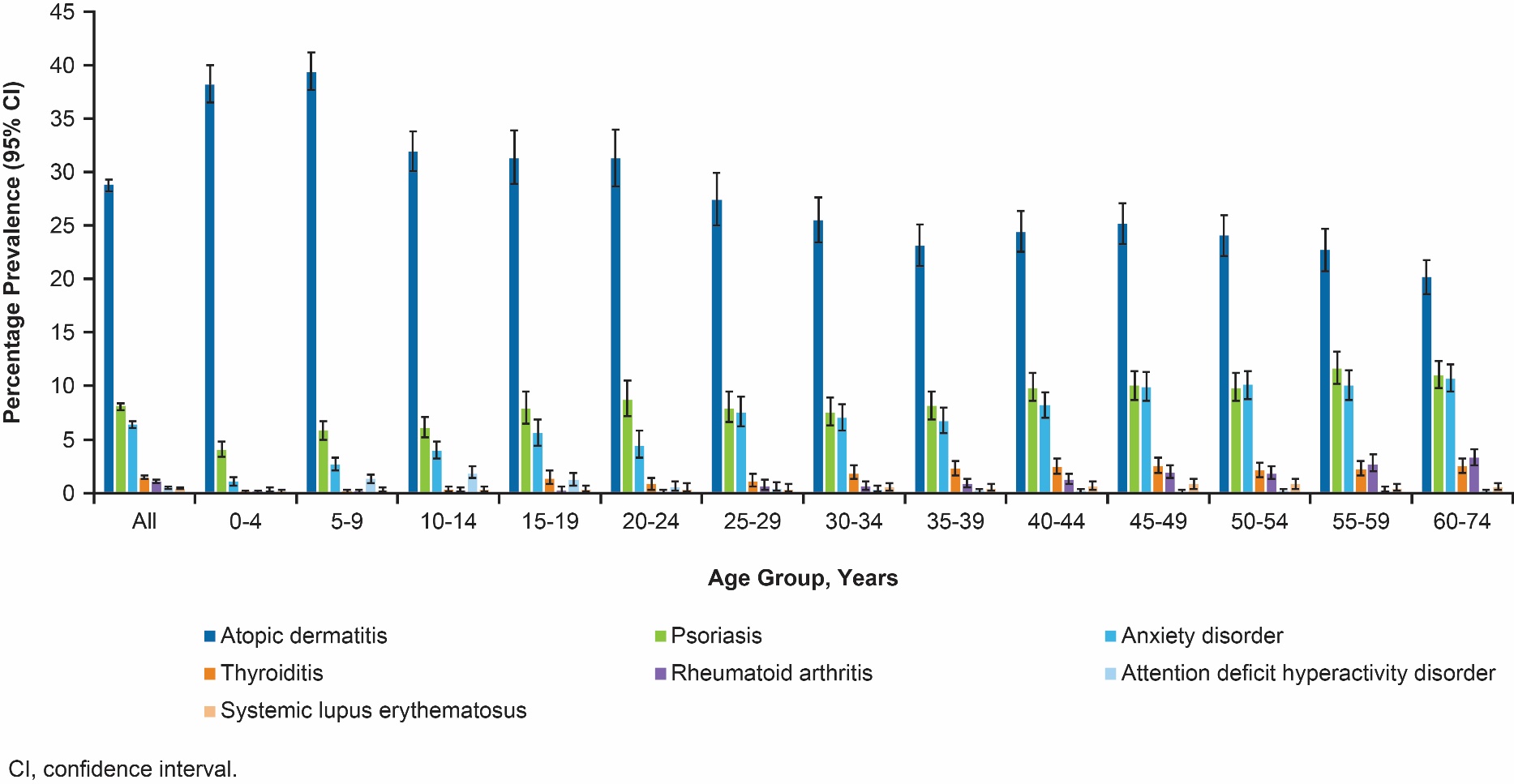


**Supporting Information Figure S5. Treatments used for vitiligo in 2022, stratified by age group (N = 26,358).**


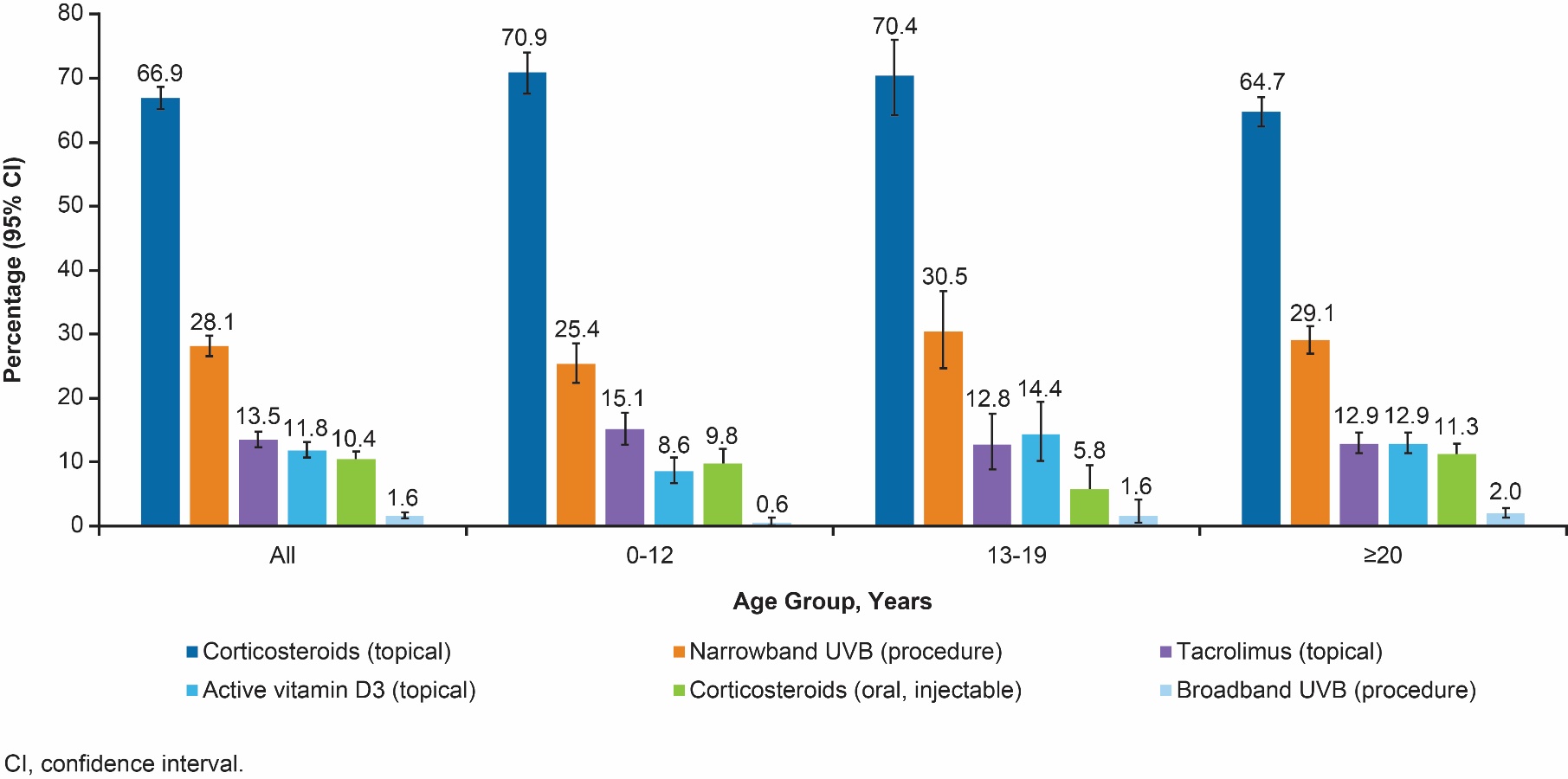

Supplement: Supplementary file 1 — Data S1. [file JDE-52-831-s001.docx]
